# The Positive and Negative Outcome of Morphine and Disulfiram Subacute Co-Administration in Rats in the Absence of Ethanol Challenge

**DOI:** 10.3390/pharmaceutics13010029

**Published:** 2020-12-26

**Authors:** Karolina Frączek, Agnieszka Kowalczyk, Martyna Pekala, Kaja Kasarello, Grażyna Sygitowicz, Dorota Sulejczak, Malgorzata Zaremba, Marek Konop, Malgorzata Frankowska, Malgorzata Filip, Magdalena Bujalska-Zadrozny, Patrycja Kleczkowska

**Affiliations:** 1 Centre for Preclinical Research (CBP), Department of Pharmacodynamics, Medical University of Warsaw, Banacha 1B, 02-097 Warsaw, Poland; Karolina.pawlik@wum.edu.pl (K.F.); kowalczykaga@op.pl (A.K.); m.pekala@nencki.edu.pl (M.P.); mbujalska@wum.edu.pl (M.B.-Z.); 2 Centre for Preclinical Research (CBP), Department of Experimental and Clinical Physiology, Medical University of Warsaw, Banacha 1B, 02-097 Warsaw, Poland; kaja.kasarello@wum.edu.pl; 3 Department of Clinical Chemistry and Laboratory Diagnostics, Medical University of Warsaw, Banacha 1, 02-097 Warsaw, Poland; gsygitowicz@poczta.onet.pl; 4 Department of Experimental Pharmacology, Mossakowski Medical Research Institute, Polish Academy of Sciences, Pawinskiego 5, 02-106 Warsaw, Poland; dots@op.pl; 5 Centre for Preclinical Research (CBP), Department of Experimental and Clinical Pharmacology, Medical University of Warsaw, Banacha 1B, 02-097 Warsaw, Poland; malgorzata.zaremba@wum.edu.pl; 6 Military Institute of Hygiene and Epidemiology, Kozielska 4, 01-163 Warsaw, Poland; 7 Centre for Preclinical Research (CBP), Department of Experimental Physiology and Pathophysiology, Medical University of Warsaw, Banacha 1B, 02-097 Warsaw, Poland; marek.konop@wum.edu.pl; 8 Laboratory of Drug Addiction Pharmacology, Institute of Pharmacology, Polish Academy of Sciences, Smętna 12, 31-343 Krakow, Poland; malafrania@poczta.onet.pl (M.F.); mal.fil@if-pan.krakow.pl (M.F.)

**Keywords:** analgesia, disulfiram, hepatotoxicity morphine, side effects

## Abstract

Recently, a well-known anti-alcohol agent, disulfiram (DSF), has gain much interest, as it was found to be effective in the treatment of cocaine abusers, thus also giving hope for patients addicted to opioids and other illicit drugs. Therefore, this study was aimed to investigate the possible outcome that might occur within the subacute co-administration of both morphine (MRF) and DSF in rats, but in the absence of ethanol challenge. As observed, intraperitoneal DSF dose-dependently enhanced MRF-mediated analgesia with the maximal efficacy at a dose of 100 mg/kg. Furthermore, MRF-induced tolerance and aggressive behavior were significantly reduced by DSF (100 mg/kg, i.p.) in comparison to MRF solely. Nonetheless, significant blood biochemical markers of hepatotoxicity were found (i.e., alteration in the levels of glutathione, blood urea nitrogen, etc.), following a combination of both drugs. Likewise, histological analysis of liver tissue revealed severe changes in the group of DSF + MRF, which includes swelling, cell death, damage to certain vessels, and hemorrhages into the liver parenchyma. Our findings indicate that DSF should be used with extreme caution, especially within the course of subacute concomitant use with MRF, as several possible side effects may take place.

## 1. Introduction

Addiction to opioids as a result of overprescribed pain medicines or being related to non-medical repeated use over time is one of the emerging international public health problems so far. Unfortunately, currently, there is no effective drug to treat opioid abuse, although detoxification can be nicely facilitated by using dose-tapered opioid agonists (mainly methadone) [1]. However, it seems that detoxification, and thus reduction in the use of the drug, in contrast to the rehabilitation of such patients, is quite complicated. Indeed, different mechanisms of therapy need to be involved, so recently stabilized patients can achieve sustained periods of drug-free living.

Recently, disulfiram (DSF), the first drug approved in the early 1950s by the US Food and Drug Administration (FDA) to treat chronic alcohol dependence (AUDs), has gained much interest as a potent drug against addiction. DSF activity is due to the ability to irreversibly inhibit the aldehyde dehydrogenase enzyme (ALDH) [2,3]. Thus, causing the disulfiram-ethanol reaction, which takes place following the consumption of ethanol. Although its mechanism of action in treating AUD is well-known, DSF has shown promise in treating cocaine addicts, both in the animal as well as human studies [4,5,6,7,8]. Indeed, the administration of DSF resulted in consecutive abstinence from cocaine. Moreover, DSF was reported to block cocaine-induced reinstatement in laboratory animals. Furthermore, DSF was found to be a useful drug candidate in opioid users [9], as well as in the treatment of non-substance-abuse disorders, such as pathological gambling [10]. However, the opposite effects were noted for DSF following cannabis smoke. The result of such a combination was euphoria, pressure of speech, and irritability [11].

Nevertheless, concerning the above findings, no data are describing DSF’s role in patients actively abusing opioids, particularly morphine (MRF). To give a tentative answer to this assumption, herein we provide both the negative and positive actions of MRF and DSF co-administration in experimental animals.

## 2. Material and Methods

### 2.1. Drugs and Reagents

Drugs used in this study were as follows: disulfiram (*N,N,N’,N*-tetraethylthiuram disulfide; DSF) (Polfa Warszawa, Poland), morphine (MRF; Polfa Warszawa, Poland), and methylcellulose (Sigma Aldrich, St. Louis, MO, USA). All drugs were freshly prepared on the day of the experiment and administered intraperitoneally (i.p.). MRF was dissolved in 0.9% NaCl, whereas DSF was suspended in 0.1% solution of methylcellulose. For all experiments, the control group received 0.1% methylcellulose. In the pilot study, MRF analgesic effective dose (i.p.) was carried out; the optimal dose was chosen to be 10 mg/kg.

### 2.2. Animals

A total of 120 male Wistar rats (Bialystok, Poland), weighing 200–250 g (Experiment 1) and 450–480 g (Experiments 2 and 3), were used in this study. Aged rats were preferred, to exclude a possible confounding bias related to the compensatory properties in the liver of a young group of animals. Animals were housed in a temperature-controlled room (temperature 22 ± 2 °C), with a 12 h light/dark cycle. Food and water were allowed throughout the study.

The animals used were randomly assigned to 8 groups (Experiment 1) or 4 groups (Experiments 2 and 3), respectively. The group size was determined based on power analysis. Groups were as follows:

Experiment 1:

(1) DSF at a dose of 25 mg/kg, i.p.
(2) DSF at a dose of 50 mg/kg, i.p.
(3) DSF at a dose of 100 mg/kg, i.p.
(4) DSF (25 mg/kg) + MRF (10 mg/kg), i.p.
(5) DSF (50 mg/kg) + MRF (10 mg/kg), i.p.
(6) DSF (100 mg/kg) + MRF (10 mg/kg), i.p.
(7) MRF (10 mg/kg), i.p.
(8) 0.1% methylcellulose (vehicle), i.p.

Experiment 2:

(1) DSF at a dose of 100 mg/kg, i.p.
(2) DSF (100 mg/kg) + MRF (10 mg/kg), i.p.
(3) MRF (10 mg/kg), i.p.
(4) 0.1% methylcellulose (vehicle), i.p.

Experiment 3:

(1) DSF at a dose of 100 mg/kg, i.p.
(2) DSF (100 mg/kg) + MRF (10 mg/kg), i.p.
(3) MRF (10 mg/kg), i.p.
(4) 0.1% methylcellulose (vehicle), i.p.

Rats from Experiment 2, in a number of 40, were further taken for additional examination (Experiment 3).

The experimenters were all blinded to treatments, apart from Experiment 3. All experimental procedures using animals complied with the policies on the care and the use of laboratory animals published in the European directive 2010/63/EU and were approved by the 2nd Local Commissions for the Care and Use of Laboratory Animals for Experimental Procedures (Permit Numbers: 12/2015, 5/2016). The entire experiment design was based on the rule of the replacement, refinement, and reduction (the 3Rs), to reduce the suffering of the animals and use the minimum number of animals.

### 2.3. Experimental Design and Procedure Used

#### 2.3.1. Experiment 1: Effect of Repeated Simultaneous Administration of Disulfiram and Morphine on Thermally Evoked Pain and Biochemical Markers of Morphine-Induced Tolerance

This part of the study was designed to demonstrate whether a simultaneous administration of various doses of DSF and MRF can result in significant enhancement of analgesia.

Both MRF (10 mg/kg, i.p.) and various doses of DSF (25, 50, and 100 mg/kg, i.p.) were given to rats for 21 consecutive days. Behavioral tests were carried out on everyday drug post-administration.

##### Tail Flick Test

Changes in nociceptive thresholds were evaluated by using thermal stimuli (tail-flick test), where a light beam was used as a painful stimulus. Briefly, the ventral surface of the tail was exposed to a radiant heat stimulus in restrained rats. A cutoff time of 15 s was employed to prevent tissue damage. Measurements were taken in triplicate, at baseline (day 1; before drug administration) and on the next days, post-injection. Measurements were done on a commercially available analgesymeter (Ugo Basile, Italy). Responses were expressed as a percentage of a maximum possible effect (%MPE), calculated according to the following formula:

[(T_1_ − T_0_)/(T_2_ − T_0_)] × 100%,

where T_0_ and T_1_ are latencies before and after drug administration, respectively, and T_2_ is the cut-off time. Drug-treated and vehicle-treated (control) groups consisted of 9 or 10 rats each.

##### Enzyme-Linked Immunosorbent Assay (ELISA)

ELISA assay was used to determine whether drugs modify intracellular signaling cascade through immediate early response c-fos gene, second messenger (cAMP), and related to cAMP response element-binding protein (CREB), as well as the mu-opioid receptors.

##### Tissue Preparation

Immediately after the last measurement session, rats were decapitated, and both spinal cord (L4–L5 segment) and specific brain regions (i.e., hippocampus and periaqueductal gray) were isolated, as these areas are highly distributed with mu-opioid receptors and were found to be functionally involved in effects resulting from prolonged opioid treatment, particularly tolerance and dependence [12,13,14].

Tissues were prepared as follows: After thawing, tissue samples were weighed, and ice-cold Phosphate-buffered saline (PBS) was added, to obtain the 10% tissue homogenates. Steel beads (5 mm Stainless Steel Beads, Qiagen, Germany) were put into the tubes; homogenates were added and were homogenized in a mechanical homogenizer (TissueLyser LT, Qiagen, Germany), at 50 Hz for 5 min. Next, homogenates were subjected to two freeze–thaw cycles, to disrupt the cell membranes. After thawing, homogenates were vortexed, steel beads were removed, and homogenates were centrifuged at 5000 rpm for 15 min (Microfuge 20, Beckman Coulter Life Science, Warsaw, Poland). Supernatants were collected for further analysis.

On the exact day of the experiment, the levels of cAMP, CREB (FineTest, BIOLIM, Gdynia, Poland), c-fos (MyBioSource, San Diego, CA, USA), and mu-opioid receptors (MyBioSource, San Diego, CA, USA) were determined via the ELISA method, according to the manufacturer’s instructions. Microplates were read, using a microplate reader (BIO-RAD Microplate Reader, Warszawa, Poland), and the absorbance (optical density value) at a wavelength of 450 nm was determined.

#### 2.3.2. Experiment 2: Effect of Disulfiram and Morphine Subacute Co-Administration on Aggressive Behavior

Aggressive behavior was measured in groups of similarly treated animals placed in a plastic cage with wire roofing. After rats’ accommodation to the environment, each animal was subacutely (17 days) given either a combination of DSF (100 mg/kg, i.p.) and MRF (10 mg/kg, i.p.), DSF solely (100 mg/kg, i.p.), MRF solely (10 mg/kg, i.p.), or 0.1% methylcellulose (control group). Measurements were performed on 1, 3, 6, 9, 13, and 17 days, immediately after drug intraperitoneal administration and included the following: (i) number of fighting episodes (e.g., striking partner with one or both forepaws, and boxing), (ii) number of bites targeted at vulnerable body parts (from mild to severe), and (iii) assumption of an aggressive posture (e.g., standing on hind paws and facing each other, or standing over). Additionally, latency to the first attack (s) and total time (for the behavioral domains, (s)) were measured. These observations were made on the day before the procedure (baseline = T_0_), as well as within 60 min drug post-injection, on the exact day. Each behavior and the total time response were pooled and presented as a total value for the group.

The recording was performed by using a SONY camera (Sony HDR-XR200, Tokyo, Japan). Additionally, on the next day, each group of animals was checked for the potential of predatory behavior occurrence. Importantly, on the 17th day of the experiment, animals were also treated with the drugs.

#### 2.3.3. Experiment 3: Examination of DSF-Induced Toxicity

For the Experiment 2 run, each rat was weighed every day, and weight changes were calculated for every group of animals. Immediately after behavioral tests (Experiment 2, day 17), rats were deeply anesthetized with isoflurane, and arterial blood samples were taken by cardiac puncture (with a 21-gauge needle; 0.8 × 40 mm; Becton Dickinson S.A., Madrid, Spain). Additionally, livers were removed, to perform histopathological examination. After that, the animals were all sacrificed.

##### Colorimetric Analysis

Blood samples from the arterial vein were collected and centrifuged, to obtain serum (18,000 rpm, 20 min; Microfuge 20, Beckman Coulter Life Science, Warsaw, Poland). Liver function was analyzed by measuring the activities of aspartate aminotransferase (AST), alanine aminotransferase (ALT), and gamma-glutamyl transferase (GGTP), as well as the concentrations of albumin (ALB), glucose (GLU), reduced glutathione (GSH), blood urea nitrogen (BUN), and urea. All of these were analyzed by using commercial diagnostic kits (Alpha-Diagnostics, Warszawa, Poland). Determination of the concentration of reduced glutathione was carried out based on the Ellman method, consisting of the reduction of 5,5-dithiobis-(2-nitrobenzoic acid) (DTNB) by thiol compounds to the colored 2-nitro-5-mercaptobenzoic acid [15].

##### Histopathological Studies

To determine the extent of hepatocyte injury, livers were assessed by histopathological observation. Briefly, formalin-fixed and cryoprotected rat livers were cut into 20 µm–thick glass-mounted sections with a cryostat (CM 1850 UV, Leica, Nussloch, Germany). After that, such a prepared section was subjected to the routine procedure of hematoxylin–eosin (H&E; hematoxylin No. 1.50174, and eosin No. 1.09844, Merck Millipore, Warsaw, Poland) staining and coverslipping with DPX (slide mounting medium, Sigma-Aldrich, Schenlldorf, Germany). Stained samples were examined and captured with a Nikon light microscope (Nikon, Tokyo, Japan) equipped with a charged-coupled device (CCD) camera and image analysis system.

**Figure 1:**
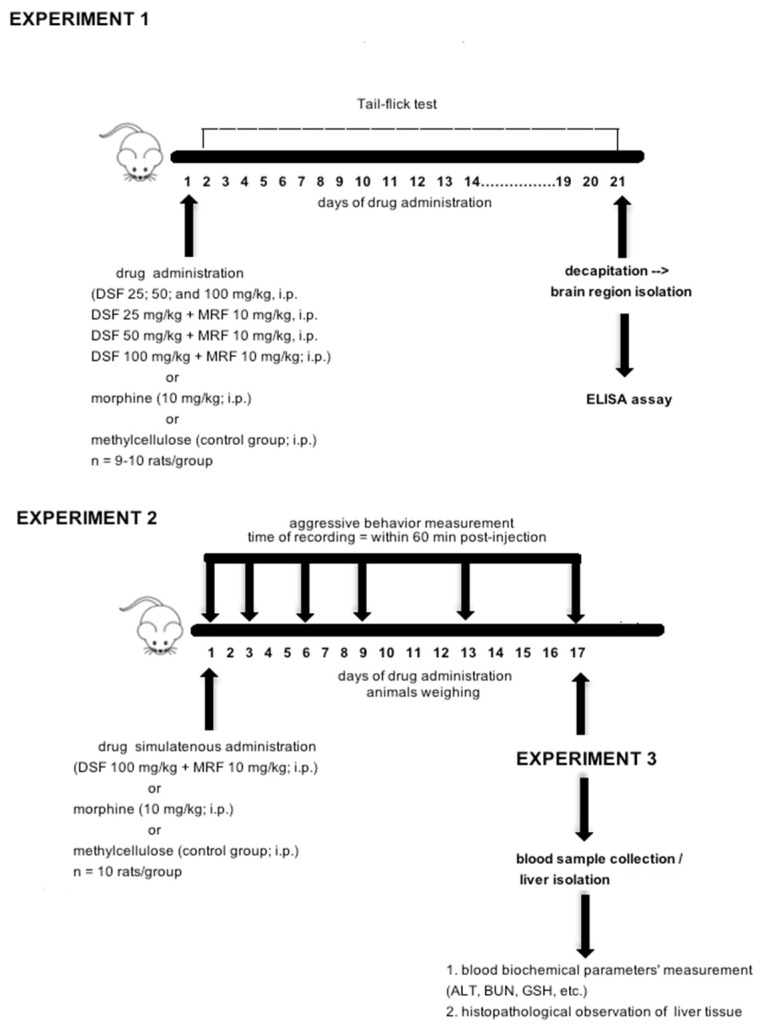
Timeline and schematic presentation of the in vivo experiments.

A figure representing all three experiments is shown below (Figure 1).

### 2.4. Statistical Analysis

The sample size was identified at 7, according to statistical power analysis. Ultimately, animal groups consisted of 8-10 rats/group. Additionally, block randomization was used to allocate animals into respective treatment groups.

Bonferroni’s post hoc test, following a two-way repeated-measures ANOVA, was used to analyze differences at individual time points (days) between experimental groups (Experiment 1, behavioral studies; Experiment 2, aggression-like behavioral parameters; and Experiment 3, body weight changes, respectively). Additionally, the area under the curve (AUC) was analyzed by using one-way ANOVA, followed by Dunnett’s post hoc test. With regards to biochemical study, statistical significance was determined by one-way ANOVA with Tukey’s post hoc test (Experiments 1 and 3). All data in the study are expressed as mean ± standard error (SEM), and a *p*-value < 0.05 was considered as statistically significant.

Statistical analysis and figures were performed with GraphPad Prism software package (version 5.00 for Mac, GraphPad Software, San Diego, CA, USA, www.graphpad.com).

## 3. Results

### 3.1. Rats Subacutely Exposed to an Escalating Dose of Disulfiram Alone Were Less Sensitive to Thermally Induced Pain Compared to Vehicle-Treated Animals

**Figure 2:**
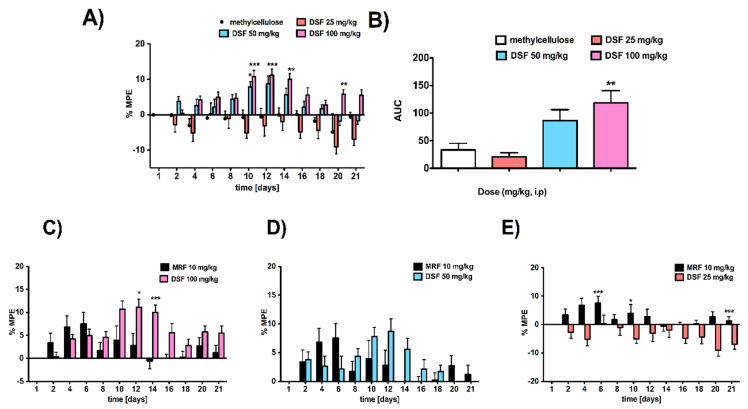
The pain-relieving effect induced by subacute (21 days) intraperitoneal administration of disulfiram (DSF). (**A**) Dose-dependent antinociceptive effect of DSF on thermally induced pain. (**B**) The area under the analgesia–time curve (AUC) after treatment with various doses of DSF vs. vehicle. (**C**,**D**,**E**) DSF (100, 50, and 25 mg/kg, respectively) effect in comparison to morphine (10 mg/kg; i.p.). Results were analyzed either with two-way ANOVA followed by Bonferroni’s post hoc test or one-way ANOVA, followed by Dunnett’s post hoc test. DSF activity was compared with a vehicle-given group (0.1% methylcellulose, i.p.). Data from every second day, starting from day 2, were omitted. Results are presented as the % of maximal possible effect (%MPE) ± SEM (9 or 10 rats per group). * *p* < 0.05, ** *p* < 0.01, and *** *p* < 0.001 indicate significant differences between DSF-treated and vehicle-treated rats (**A**,**B**). * *p* < 0.05 and *** *p* < 0.001 indicate a significant difference between morphine (MRF)- and DSF-treated animals (100 mg/kg) on 12th and 14th day of repeated once-daily intraperitoneal administration (**C**), respectively. * *p* < 0.05 and *** *p* < 0.001 indicate a significant difference between MRF- and DSF-treated animals (25 mg/kg) on 6th, 10th, and 21st day of repeated once-daily intraperitoneal administration (**E**), respectively. Rats treated with methylcellulose served as a control group; n = 10 animals (male Wistar rats)/group.

Repeated intraperitoneal (i.p.) administration of DSF exerted a dose- [F_3,36_ = 9.84; *p* < 0.0001, n = 9–10] and time-dependent [F_11,396_ = 7.86; *p* < 0.0001, n = 9–10] pain-relieving effect (Figure 2A). Compared with the effect induced by the vehicle, the lowest dose of DSF (25 mg/kg, i.p.) resulted in hyperalgesia, starting from the MPE value equal −2.77 ± 1.90% (day 2) up to −9.04 ± 2.05% on the 20th day of the experiment (Figure 2A). Meanwhile, by increasing the dose of DSF to 50 or 100 mg/kg, the strongest effect and thus the maximal activity was reached within 10-14 days of the experiment. Indeed, for the dose of DSF equal to 50 mg/kg, the MPE = 8.69 ± 2.23% and 5.59 ± 1.89%, reported on days 12 and 14, respectively. Additionally, the maximal possible effect observed for the dose of 100 mg/kg (i.p.) on days 12 and 14 was equal, at 11.16 ± 1.76% and 9.49 ± 1.64%, respectively. Importantly, there were no significant differences in the effect between both doses (i.e., 50 and 100 mg/kg). Since ED_50_ represents a dose required to achieve 50% of the maximal effect, there was no possibility to produce the ED_50_ value for drugs (DSF and/or DSF + MRF) administered intraperitoneally.

Interestingly, there were also no significant differences between the dose of 50 mg/kg DSF (i.p.) and MRF administered in the same manner, at a dose of 10 mg/kg (Figure 2D). Meanwhile DSF at a dose of 25 mg/kg did not produce analgesia (Figure 2E). Only 100 mg/kg of DSF exerted a significantly stronger analgesia (*p* < 0.05) than MRF (Figure 2C). This, however, was noted on the 12th and 14th day of the experiment (significant effect of time [F_11,187_ = 5.38, *p* < 0.0001]) (Figure 2C). Furthermore, despite quite similar responses exerted by these two pharmacologically distinct drugs (Figure 2C), an observable shift in time in terms of their antinociceptive action was noticed. In fact, MRF started to act from the very beginning of the experiment, exerting the maximal effect on day 6 (MPE = 7.54 ± 2.48%) and then slowly decreased. On the contrary, in the case of DSF, its small effect can be observed on the 4th day (4.19 ± 1.46%) of the experiment and appeared to be at the same level of MPE ≈ 4.5% until the five consecutive days. After that, within the period when MRF-induced effect is found to be going markedly down (from day 9 to 14), DSF showed its highest effectiveness in pain relief.

### 3.2. Analgesic Response to Morphine Was Affected by the Addition of Disulfiram in a Dose-Dependent Manner

**Figure 3:**
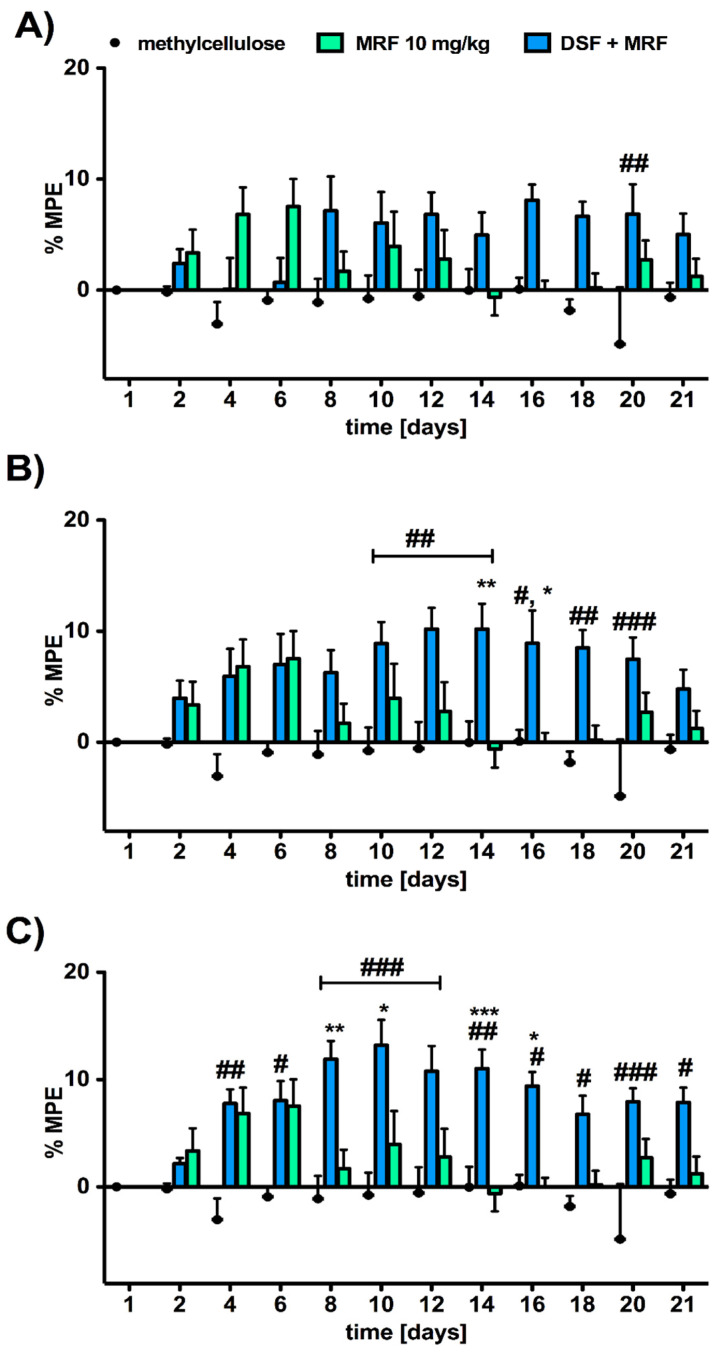
Changes in thermal pain threshold following 21 consecutive days of intraperitoneal co-administration of (**A**) DSF (25 mg/kg) and MRF (10 mg/kg); (**B**) DSF (50 mg/kg) and MRF (10 mg/kg); and (**C**) DSF (100 mg/kg) and MRF (10 mg/kg), assessed in the radiant heat tail-flick test. Analgesic effects were recorded each day, before drugs’ injections. Two-way ANOVA with Bonferroni’s post hoc test was used to determine differences between treatment groups at each time point (days). * *p* < 0.05, ** *p* < 0.01, and *** *p* < 0.001 indicate a significant difference compared with MRF; ^#^ *p* < 0.05, ^##^ *p* < 0.01, and ^###^ *p* < 0.001 vs. vehicle-treated animals; n = 9 or 10 animals (male Wistar rats)/group.

We observed that the MRF-induced analgesic effect was significantly enhanced and prolonged after the addition of DSF, especially at doses of 50 (significant effect of treatment [F_2,26_ = 8.90; *p* < 0.011] and time [F_11,286_ = 1.81; *p* = 0.0515]; Figure 3B) and 100 mg/kg (significant effect of treatment [F_2,26_ = 17.36; *p* < 0.0001] and time [F_11,286_ = 2.86; *p* = 0.0014]; Figure 3C), respectively. Although for every combination the maximal analgesic response was found to be weak, as the MPE value did not exceed 20%, DSF’s beneficial impact was noticed. Indeed, as the maximal MRF-induced pain-relieving effect was reached on day 10, with the value of 3.95 ± 2.12%, a daily co-administration of DSF (100 mg/kg) and MRF resulted in the highest level of MPE = 13.20 ± 2.37% on the same day (*p* < 0.001). Intriguingly, the highest dose of DSF (100 mg/kg), combined with MRF (Figure 3C), appeared to possess a diverse pharmacodynamics profile from a combination with 50 mg/kg of DSF (Figure 3B). When administering a mixture containing the lower dose of DSF, a nice bell-shaped curve was observed, with the maximal value of MPE equal 10.20 ± 2.27%. Meanwhile, the co-administration of MRF and DSF (100 mg/kg; Figure 3C) produced the strongest activity four days earlier than the previous one. Moreover, the analgesia mediated by such a combination (Figure 3C) seemed not to be reduced significantly within the next consecutive days, as it was maintained at the constant level of MPE = 8–11%.

### 3.3. Subacute Administration of Disulfiram and Morphine Did Not Alter c-fos, cAMP, and Mu-Opioid Receptor Levels except Transcription Factor (CREB) Protein in the Periaqueductal Gray (PAG), Hippocampus, and Spinal Cord

**Figure 4:**
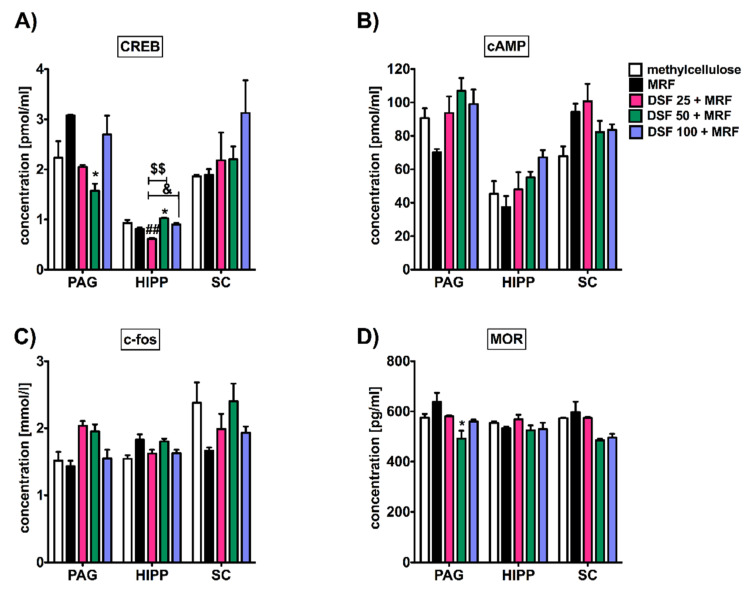
Changes observed in (**A**) CREB, (**B**) cAMP, (**C**) c-fos, and (**D**) mu-opioid receptor (MOR) levels in the brain and spinal cord tissues after DSF and MRF subacute intraperitoneal co-administration in comparison to morphine-treated rats by using the same paradigm. PAG, periaqueductal gray; HIPP, hippocampus; SC, spinal cord (spinal level); One-way ANOVA with Tukey’s post hoc test revealed differences in CREB levels in groups of: MRF vs. DSF 50 + MRF (* *p* < 0.05), methylcellulose vs. DSF 25 + MRF (^##^ *p* < 0.01), DSF 25 + MRF vs. DSF 50 + MRF (^$$^ *p* < 0.01), and DSF 25 + MRF vs. DSF 100 + MRF (^&^ *p* < 0.05), respectively. Additionally, a significant difference (* *p* < 0.05) was shown in mu-opioid receptor concentration in the PAG region between MRF and DSF 50 + MRF; n = 9 or 10 animals (male Wistar rats)/group.

As shown in Figure 3, significant changes between MRF and a mixture with DSF-treated animals particularly affected the CREB protein level in both PAG and the hippocampus (Figure 4A). However, only in the hippocampus were these changes reported for various doses of DSF in combination with MRF (i.e., 25, 50, and 100 mg/kg). Furthermore, DSF at a lowest dose of 25 mg/kg (i.p.) resulted in a reduced CREB protein level, in comparison to the control, ranging from values of 0.812 ± 0.027 pmol/mL (methylcellulose) to 0.616 ± 0.015 pmol/mL (DSF 25 + MRF) (95% CI, 0.12 to 0.51; *p* < 0.01; control vs. DSF 25 + MRF), respectively. By increasing the dose of DSF up to 50 mg/kg, CREB levels increased to the value equal 1.027 ± 0.013 pmol/mL, whereas the use of the highest DSF dose (100 mg/kg) recovered CREB levels to normal values reported for MRF.

Similar to the above, the addition of escalating doses of DSF did not alter the level of cAMP (Figure 4B), c-fos (Figure 4C), or mu-opioid receptors (Figure 4D).

### 3.4. Repeated Simultaneous Administration of Disulfiram and Morphine at the Highest Dose Tested Affected Morphine-Induced Aggressive Response in Rats

In this study, we used the highest dose (100 mg/kg) of DSF, with the most significant analgesic response noticed in the thermally evoked pain analysis (please see Figure 2 and Figure 3). This choice was also based on several studies and indicated that such an i.p. DSF dose is found to be a close functional match to those used in humans. This dose is likely to produce both behavioral and neurochemical effects [16,17], as well as significantly affect dopamine β-hydroxylase (DBH) activity [18]. Moreover, as it was shown in studies strictly related to cocaine, in rats, the dose of 100 mg/kg of DSF (and higher) was sufficient to block cocaine-induced reinstatement of cocaine-seeking behavior [18].

**Figure 5:**
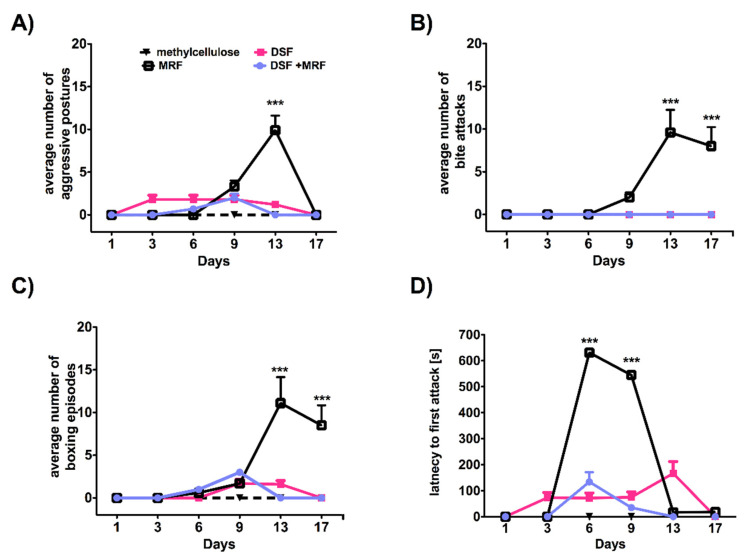
Aggression-like behavioral parameters recorded for rats intraperitoneally treated with DSF-, MRF- or its mixture. (**A**) Average number of aggressive postures; (**B**) Average number of bite attacks; (**C**) Average number of boxing episodes; and (**D**) Latency to first attack. Aggressive interactions were recorded on days 1, 3, 6, 9, 13, and 17, for 60 min, immediately after drug administration. Two-way ANOVA, followed by Bonferroni’s post hoc test, revealed significant differences for MRF vs. DSF + MRF (***). Although differences between MRF and methylcellulose, DSF and methylcellulose, DSF + MRF vs. methylcellulose, and DSF vs. DSF + MRF occurred in each experiment, they are not shown in the figure; n = 10 animals (male Wistar rats)/group.

As shown in Figure 5, rats administered MRF were the most aggressive among every tested group. Importantly, the highest number of aggressive incidents was usually observed on the 13th day (Figure 5). Compared to MRF alone, co-injection (MRF + DSF) significantly reduced MRF-evoked aggression. Such a phenomenon was noticed for the following parameters: aggressive posture [F_5,180_ = 32.69; *p* < 0.0001 and F_3,36_ = 14.52; *p* < 0.0001; Figure 5A], the number of bite attacks [F_5,180_ = 12.82; *p* < 0.0001 and F_3,36_ = 13.42; *p* < 0.0001; Figure 5B], the number of boxing episodes [F_5,180_ = 14.53; *p* < 0.0001 and F_3,36_ = 10.70; *p* < 0.0001; Figure 5C], and finally for the latency to first attack [F_5,180_ = 247.15; *p* < 0.0001 and F_3,36_ = 84.49; *p* < 0.0001; Figure 5D].

### 3.5. Morphine and Disulfiram Co-Administration Significantly Augmented Disulfiram-Induced Hepatic Failure

**Figure 6:**
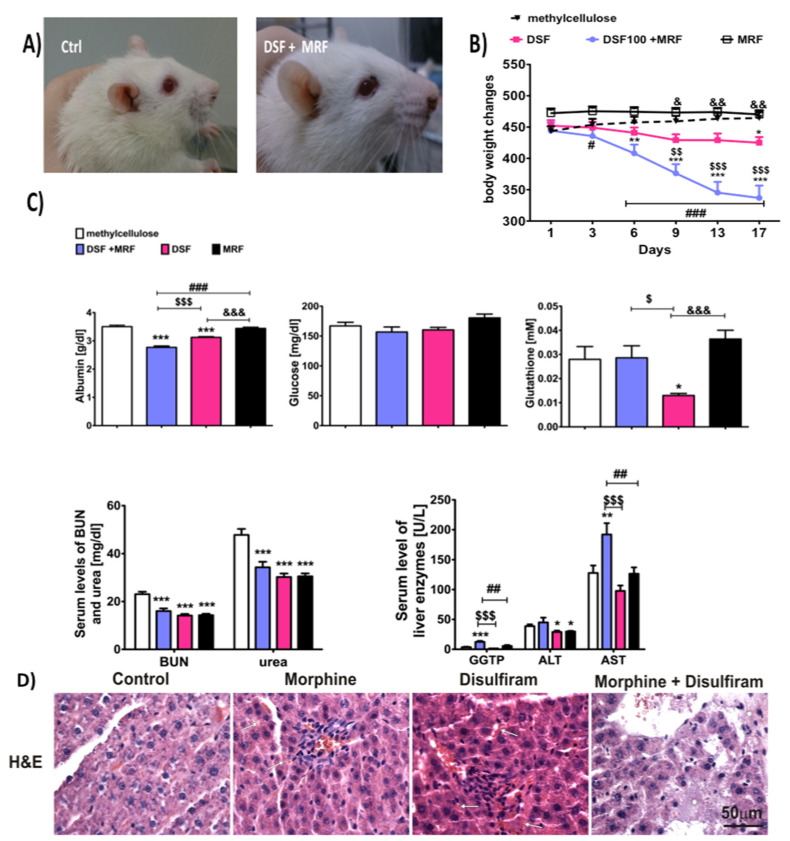
Pictures of rats (**A**); total body weight changes (**B**); changes in AST, ALT, GGTP, glucose, albumin, glutathione (GSH), urea, and blood urea nitrogen (BUN) concentrations in serum samples from arterial blood (**C**); and histopathological changes in the liver (**D**), as a consequence of DFS and MRF repeated daily intraperitoneal co-injections. Histopathological examination was performed on liver tissue samples, while the levels of liver enzymes and other agents were determined from aortic blood samples. One-way ANOVA with Tukey’s post hoc test (panel **B** and **C**) was used to determine differences between treatment groups. Statistical differences were considered significant at *p* < 0.05, *p* < 0.01, and *p* < 0.001, respectively. * For drug-treated groups (DSF, MRF, or DSF + MRF) vs. vehicle (methylcellulose, control); # for DSF + MRF vs. MRF; $ for DSF + MRF vs. DSF; and & for DSF vs. MRF. × 400; n = 8–10 animals (male Wistar rats)/group.

At the beginning of the experiment, all rats were healthy and behaved normally. Within the next several days, rats given DSF + MRF lost weight (Figure 6B). Moreover, starting from day nine of the experiment, the above group revealed significant sleepiness, powerlessness, and sometimes shivers. Additionally, porphyrin secretion (chromodacryorrhea) was observed, as colored crusts were present around the eyes and nostrils (Figure 6A). Ultimately, two rats died on days 16 and 17, respectively.

With regards to liver functionality (Figure 6C), concomitant subacute administration of DSF and MRF at a dose of 100 mg/kg and 10 mg/kg (i.p.), respectively, caused a significant reduction in albumin level (2.77 ± 0.05 g/dL), in comparison to methylcellulose- (control; 3.51 ± 0.05 g/dL; 95% CI, 0.57 to 0.90; mean difference, 0.735; *p* < 0.001), DSF- (3.13 ± 0.03 g/dL; 95% CI, −0.52 to −0.19; *p* < 0.001) and MRF- (3.44 ± 0.04 g/dL; 95% CI, −0.84 to −0.50; mean difference, −0.355; *p* < 0.001) treated groups. Moreover, in rats that were injected with a single DSF, and compared with the control, the loss of albumin was revealed, while MRF solely did not produce such a phenomenon. Similarly, a marked increase was indicated for AST (DSF + MRF vs. methylcellulose: 192.07 ± 18.96 U/L; 95% CI, −115 to −13.7; mean difference −64.2; *p* < 0.01) and GGTP activity (DSF + MRF vs. methylcellulose: 12.52 ± 1.57 U/L; 95% CI, −13.9 to −3.97; mean difference −8.92; *p* < 0.001), in comparison to control animals (AST: 127.83 ± 12.48 U/L and GGTP: 3.60 ± 0.72 U/L, respectively). Importantly, there were no significant changes between animals given with DSF or MRF alone, or for the control group (*p >* 0.05).

Although statistical differences between both the DSF + MRF and control group were observed for most of the liver enzyme (except from ALT: 33.75 ± 3.06 U/L vs. 38.77 ± 2.98 U/L; 95% CI, −4.91 to 14.9; mean difference, 5.02; *p >* 0.05), both the serum blood urea nitrogen (BUN) and urea levels were decreased in every drug-treated group (*p* < 0.001), in comparison to the control. However, neither DSF-, MRF-, nor DSF + MRF–treated animals showed a level’s dissimilarity, as the concentrations for BUN and urea were equal, at 14–16 mg/dL and 30–34 mg/dL, respectively (Figure 6C). Furthermore, the lack of differences was reported for glutathione (*p* > 0.05 for DSF + MRF vs. control). However, compared to the control animals, rats with DSF solely demonstrated almost a half reduction of its concentration (*p* < 0.05).

The histopathological assessment indicated severe changes in the liver in the group co-treated with DSF + MRF, mostly swelling, cell death, damage to certain vessels, and overall disturbance of liver structure (Figure 6D). The same pathological changes were not observed in the control, MRF, or DSF group. Minor liver dysfunction was noticed in DSF animals, particularly marked congestion (typical for hyperemia); however, in most of the cells, the morphology was normal. Similarly, MRF did not cause liver damage.

## 4. Discussion

### 4.1. Disulfiram Pain-Relieving Effect and Its Impact on Analgesia and Morphine Tolerance Development

This paper demonstrates disulfiram (DSF) as a potent factor in the enhancement of morphine (MRF)-induced analgesia; it dose-dependently suppresses pain nociception when co-administered intraperitoneally. More importantly, a simultaneous repeated administration of both compounds resulted in the extension of the pain-relieving effect of MRF, as the attenuation of thermal nociception remained on the same level of MPE, at approximately 15% (Figure 3C).

To date, little is known about DSF’s anti-pain potency, as only limited studies, with conflicting data, were published.

In one of the papers, Rahman et al. [19] revealed that chronic administration of DSF resulted in a significant increase of latencies in response to thermally induced nociception that returned to baseline values following the cessation of the drug. On the contrary, several case studies, including one recently presented by Tran et al. [20], reported DSF-induced neuropathy [21,22]. Nonetheless, it has been reported that DSF diminished the pain-related substance P (SP) level in the spinal cord [23]. Given the inhibitory action of DSF on SP and well-known opioid-related reduction of SP release on presynaptic neuronal terminals, it may explain the analgesic (synergy) effect related to concomitant use of DSF and MRF in our study (Figure 3). Apart from SP, DSF was also found to affect ALDH (aldehyde dehydrogenases); thus, it induces changes in the metabolite patterns of dopamine (DA), norepinephrine (NE), and 5-hydroxytryptamine (5-HT). Therefore, for instance, if the level of 5-HT is also increased, a pain-relieving effect is noted, indicating another specific mechanism of DSF in pain treatment. Nevertheless, additional experiments need to be performed to confirm such a hypothesis.

In our experiments, as presented in Figure 2C, on day eight, DSF acted stronger than MRF. However, within the first days of morphine administration, this drug was stronger than DSF. Nonetheless, after this period, a tolerance development to MRF analgesia occurred. We think that the time of measurements plays a crucial role here. In fact, the tail-flick test was performed every 24 h post-injection. Therefore, the observed effect, in the case of MRF, was possibly due to the accumulation of M-3-G (morphine-3-glucuronide), which is devoid of any analgesic effect. However, by activating the Toll-like 4 receptors, it is responsible for anti-analgesic and hyperalgesic effects.

Similar effects may take place in the case of DSF, but of the opposite nature, eventually resulting in metabolites showing analgesic properties.

Although the delay tolerance exerted by a co-administration of DSF and MRF may reflect the DSF disrupting mechanism of DA metabolism and homeostasis, the analgesic potency of DSF solely is yet to be fully discovered.

### 4.2. Disulfiram Impact on Morphine-Induced Tolerance Markers

Morphine tolerance and dependence influence levels of cAMP, c-fos, opioid receptors, CREB, and other elements in the brain. Moreover, for instance, CREB, as well as c-fos, are well-known markers of morphine-induced tolerance (but not other opiates/opioids), and they are implicated in brain actions of drugs of abuse.

Surprisingly, along with the behavioral response, we noticed changes at the biochemical levels in some markers of MRF tolerance. Concomitant injection of both DSF and MRF resulted in marked modifications for CREB protein expression, particularly in the hippocampus (Figure 4A), which seems to be valuable information. In fact, the hippocampus is known for its interactions with nucleus accumbens (NAc), a brain region important for the reinforcing actions of many types of abused substances. Moreover, chronic morphine administration is found to reduce the total CREB level, and the result is considered to be the compensatory mechanism to the increase in the CREB phosphorylation caused by upregulation of the PKA pathway. Nonetheless, it is known that opioids could affect various intracellular mediator systems: inhibitory, which is connected with cAMP, and stimulatory, which involves the Ca^2+/^PKC pathway and affects CREB in different phases of drug action [24]. Given the above, we can hypothesize that DSF may strongly influence the rewarding action of morphine via the reduction of its properties of compulsive drug-seeking behavior, which is in line with our studies recently performed (data not published). However, DSF + MRF co-administration did not alter the level of other markers (cAMP and c-fos) (Figure 4B,C), as well as mu-opioid receptors, since DSF does not affect brain opioid receptors directly [25].

### 4.3. Disulfiram Impact on Morphine-Induced Aggressive Response

Aggressive behaviors can be associated with drug-abuse-related neurochemical brain changes. Mostly, repeated consumption of several psychoactive substances and/or drugs, as well as rapid cessation from such, may lead to significant changes in mood.

MRF is known to induce aggression and violent behavior [26], not only because of its cessation but also while chronic treatment [27,28,29]. The same phenomenon was also observed in our study (Figure 5). Similar to MRF, DSF-treated animals exhibited aggression towards each other, but to a lesser extent. This could be explained by the fact that aggressive behavior may be defined as an oxidative-stress-response reaction. DSF is a well-known inhibitor of an antioxidant enzyme, SOD1 (a copper–zinc superoxide dismutase), which plays a role in oxidative stress. This, in turn, may be associated with exaggerated animal aggressiveness, as it was suggested by many groups. In fact, it was reported that mice deficient with SOD1, and thus showing an elevation in oxidative damage, expressed overt aggression [30]. Moreover, Constantini et al. [31] presented that aggressive mice were characterized by lower antioxidant capacity in comparison with non-aggressive ones. In addition, DSF significantly reduced glutathione (GSH) concentration (Figure 6C), another critical protector of oxidative damage. A decrease in GSH level, together with an escalation of aggression was, however, absent following long-term exposure to DSF + MRF (Figure 5 and Figure 6C).

Additionally, numerous studies about spontaneous aggression suggest the critical role of both dopaminergic and noradrenergic transmissions. The recent findings published by Dvoto et al. [32] have shown that DSF stimulates DA release.

Extreme aggression followed by substantial DA release should have been expected in animals treated with a combination of both drugs. Nevertheless, in this case, both the in vivo and ex vivo studies are inconsistent, as this behavioral response did not take place, particularly in old animals (weighing 450-480 g; Experiments 2 and 3). Given that, within the first experiment, rats weighing 200-250 g were reported to behave aggressively to each other (data not shown), the repeated DSF + MRF administration paradigm was applied to confirm similar behavior (Experiment 2). The obtained results from Experiment 2, especially the lack of aggression in DSF + MRF rats, may result from the toxicity of combined drugs, but also indicate that age can be an important confounding factor for impaired resistance to the toxic effect of DSF and MRF.

According to other studies, the aggressive behavior was attenuated by beta-hydroxylase (DBH) reduction, following DSF administration [33]; it also corresponded with NE rather than DA [34]. In addition, our preliminary study showed augmented DA immunoreactivity (IR) in the cerebral cortex of rats, following MRF administration; however, co-administration with DSF induced an opposite effect (data not shown). Such a phenomenon needs further investigation, and we plan a more detailed biochemical study in this field.

### 4.4. The Concomitant Therapy and Disulfiram-Induced Hepatic Failure

DSF was found to be critically involved in hepatotoxicity [35,36]. In human reports, DSF-induced hepatic failure and, in some cases, fatal hepatitis, resulting in transplantation or death [25]. Based on the Summary of Product Characteristics (SPC) for DSF alone, in humans, hepatotoxicity is relatively rare among other reported side effects.

In the present study, we have also observed a kind of association between subacute simultaneous DSF + MRF intake and weight loss, one of the main symptoms of DSF-induced hepatic impairment. This further prompted us to examine liver function in rats. As a result, we found numerous histopathological changes in the liver in the group co-treated (DSF + MRF), including swelling, cell death, and damage to certain vessels (Figure 6D). We also presume that hepatic oxidative stress or impaired liver function initiated a compensatory mechanism manifest in the liver by the higher level of glutathione (GSH), which was noticed in the group co-treated with DSF + MRF. These results were quite unexpected, as DSF causes significant depletion of GSH in the liver and other tissues (i.e., brain), rather than its accumulation and compensatory protection [37,38]. Similarly, there are no significant differences in GSH between MRF- and control-treated groups (Figure 6C). We presume that a diminished level of GSH in the concomitant exposure to MRF and DSF may be related to boost metabolic activity and intense thiol groups’ intake in the liver. It is worth noting that GSH is a powerful antioxidant and protein modulator involved in the hepatocellular reaction to toxic compounds [39]. The lower (for DSF) or non-significant change (for MRF) in the concentration of glutathione after the action of the observed drugs may also be caused by the disturbance of its biosynthesis. Moreover, both drugs cause changes in redox potential [40].

However, this may be implicated with a completely different mechanism(s) than when both drugs are investigated separately; the mechanism may possibly also not be related to ROS. Regulation of transcription of genes encoding enzymes (such as antioxidant response element, ARE) involved in antioxidant defense may be one of the examples. This enzyme is an important DNA fragment essential for basal expression and induction of enzymes that metabolize certain medications [41]. Nonetheless, to investigate the exact mechanism of liver damage in more detail, in subsequent studies, we intend to determine the GSH/GSSG ratio, which will allow us to assess the oxidation-reduction state of liver cells in all treatment regimes.

By analyzing other biochemical serum parameters, we may come to the conclusion that MRF and/or DSF may directly affect the liver and kidney functioning; however, results were inconclusive. For example, when we investigated BUN and urea levels, neither DSF nor MRF administered solely or simultaneously caused an increased in BUN or urea level changes (Figure 6C). This was in contrast with several papers that reported MRF to increase BUN both in mice and rats, in comparison to non-treated animals [42,43]. However, the appearance of such effects was generally observed for MRF at high doses (from 12 mg/kg i.p. for rats [42] to 30 mg/kg i.p. twice daily for mice [43]) or in the long-term administration (e.g., 30 days) [42]. On the contrary, DSF can significantly reduce BUN levels, as shown by Khairnar et al. [44], and the same action was observed in our study. Likewise, MRF + DSF co-administration did not reduce BUN concentration, suggesting a lack of pharmacodynamics interaction.

It was also reported that the level of serum glucose can be altered either by acute or chronic MRF administration, but the effect is both time- and dose-dependent. For instance, the low dose of MRF decreases the plasma epinephrine and leads to hypoglycemia, while high doses result in an increase in glucose production by the liver. However, usually, the effect was not statistically significant [45]. This observation is in accordance with our study (Figure 6C), as we reported MRF to slightly increase blood glucose levels. On the contrary, DSF was found to contribute to lower glucose levels in animals with type 2 diabetes [46]. Similarly, in our study, the DSF administration resulted in a non-significant decrease. Even the simultaneous injection of both drugs did not alter the level of glucose, suggesting inhibitory interaction between both tested drugs.

Insights from our study revealed that co-administration with MRF may dramatically change the safety profile for DSF, which seems to be highly important, especially for drug abusers mixing medications without knowing.

Surprisingly, in our study, MRF alone did not affect liver activity; these results are in contrast with several reports indicating that MRF has a hepatotoxic potency [47,48]. We presume that such a phenomenon could be elicited by using a small dose of MRF (10 mg/kg i.p.) in the study.

## 5. Conclusions

To conclude, the addition of DSF at 100 mg/kg (i.p.) dose to MRF has many advantages in our study, including enhancement of MRF-induced analgesia, delayed opioid-related tolerance, and reduction of aggressive behavior. However, the crucial limitation for such co-administration resulted in serious side effects of hepatotoxicity in rats and probable risk of liver injury. Of note, DSF, especially a dose of 100 mg/kg (i.p.), is widely used and is consequently suggested to be effective in cocaine abuse, and possibly other psychoactive substances.

The results of the present study are promising and should be taken into serious consideration regarding any use of DSF in addicts; however, more extensive toxicological studies should be performed.

## Data Availability

Not applicable.

## References

[1] Whelan P.J., Remski K. (2012). Buprenorphine vs. methadone treatment: A review of evidence in both developed and developing worlds. J. Neurosci. Rural. Pract..

[2] Amador E., Gazdar A. (1967). Sudden death during disulfiram–alcohol reaction. Q. J. Stud. Alcohol.

[3] Gessner P.K., Gessner T., Genner P.K., Gedddner T. (1992). Relevant physical and chemical properties. Disulfiram and Its Metabolite, Diethyldithio Carbamate.

[4] Gaval-Cruz M., Weinshenker D. (2009). Mechanism of disulfiram-induced cocaine abstinence: Antabuse and cocaine relaps. Mol. Interv..

[5] Carroll K., Ziedonis D., O’Malley S., McCance-Katez E., Gordon L., Rounsaville B. (1993). Pharmacologic interventions for alcohol-and-cocaine-abusing individuals. Am. J. Addict..

[6] Grassi M.C., Cioce A.M., Giudici F.D., Antonilli L., Nencini P. (2007). Short-term efficacy of disulfiram or naltrexone in reducing positive urinanalysis for both cocaine and cocaethylene in cocaine abusers: A pilot study. Pharmacol. Res..

[7] Maj J., Przegalinski E., Wielosz M. (1968). Disulfiram and the drug-induced effects on motility. J. Pharm. Pharmacol..

[8] Nich C., McCance-Katz E.F., Petrakis I.L., Cubells J.F., Rounsaville B.J., Carroll K.M. (2004). Sex differences in cocaine-dependent individuals‘ response to disulfiram treatment. Addict. Behav..

[9] de Corde A., Krząścik P., Wolińska R., Kleczkowska P., Filip M., Bujalska-Zadrożny M. (2018). Disulfiram attenuates morphine or methadone withdrawal syndrome in mice. Behav. Pharmacol..

[10] Reuter J., Raedler T., Rose M., Hand I., Glasher J., Buchel C. (2005). Pathological gambling is linked to reduced activation of the mesolimbic reward system. Nat. Neurosci..

[11] Lacoursiere R.B., Swatek R. (1983). Adverse interaction between disulfiram and marijuana: A case report. Am. J. Psychiatry.

[12] Nestler E.J. (2002). Common molecular and cellular substrates of addiction and memory. Neurobiol. Learn. Mem..

[13] Williams J.T., Christie M.J., Manzoni O. (2001). Cellular and synaptic adaptations mediating opioid dependence. Physiol. Rev..

[14] Yang H.Y., Pu X.P. (2009). Chronic morphine administration induces over-expression of aldolase C with reduction of CREB phosphorylation in the mouse hippocampus. Eur. J. Pharmacol..

[15] Sedlak J., Lindsay R.H. (1968). Estimation of total, protein-bound, and nonprotein sulfhydryl groups in tissue with Ellman’s reagent. Anal. Biochem..

[16] Major L.F., Lerner P., Ballenger J.C., Brown G.L., Goodwin F.K., Lovenberg W. (1979). Dopamine-beta-hydroxylase in the cerebrospinal fluid: Relationship to disulfiram-induced psychosis. Biol. Psychiatry.

[17] Paradisi R., Grossi G., Pintore A., Venturoli S., Porcu E., Capelli M. (1991). Evidence for a pathological reduction in brain dopamine metabolism in idiopathic hyperprolactinemia. Acta Endocrinol..

[18] Schroeder J.P., Cooper D.A., Schank J.R., Lyle M.A., Gaval-Cruz M., Ogbonmwan Y.E. (2010). Disulfiram attenuates drug-primed reinstatement of cocaine seeking via inhibition of dopamine β-hydroxylase. Neuropsychopharmacol.

[19] Rahman M.A., Grunberg N.E., Mueller G.P. (1997). Disulfiram causes sustained behavioral and biochemical effects in rats. Pharmacol. Biochem. Behav..

[20] Tran A.T., Rison R.A., Beydoun S.R. (2016). Disulfiram neuroapthy: Two case reports. J. Med. Case Rep..

[21] Bradley W.G., Hewer R.L. (1966). Peripheral neuropathy due to disulfiram. Br. Med. J..

[22] Gardner-Thorpe C., Benjamin S. (1971). Peripheral neuropathy after disulfiram administration. J. Neurol. Neurosurg. Psychiat..

[23] Marchand J.E., Hershman K., Kumar M.S.A., Tjompson M.L., Kream R.M. (1990). Disulfiram administration affects substance P-like immunoreactive and monoaminergic neural systems in rodent brain. J. Biol. Chem..

[24] Bilecki W., Przewłocki R. (2000). Effect of opioids on Ca2+/cAMP responsive element binding protein. Acta Neurobiol. Exp. Wars.

[25] Center for Substance Abuse Treatment (2009). Incorporating Alcohol Pharmacotherapies Into Medical Practice. Treatment Improvement Protocol (TIP) Series 49. HHS Publication No. (SMA) 09-4380.

[26] Berman M., Taylor S., Merged B. (1993). Morphine and human aggression. Addict. Behav..

[27] Kantak K., Miczek K. (1998). Social, motor, and autonomic signs of morphine withdrawal; differential sensitivities to catecholaminergic drugs. Psychopharmacology.

[28] Kanui T.I., Hole K. (1990). Morphine induces aggression but not analgesia in the naked mole-rat (Heterocephalus glaber). Comp. Biochem. Physiol. Part C Comp. Pharmacol..

[29] Sukhotina I.A. (2001). Morphine withdrawal-facilitated aggression is attenuatedby morphine-conditioned stimuli. Pharmacol. Biochem. Behav..

[30] Garrat M., Brooks R.C. (2015). A genetic reduction in antioxidant function causes elevated aggression in mice. J. Exp. Biol..

[31] Constantini D., Carere C., Caramaschi D., Koolhaas J.M. (2008). Aggressive and non-aggressive personalities differ in oxidative status in selected lines of mice (Mus musculus). Biol. Lett..

[32] Dvoto P., Flore G., Saba P., Cadeddu R., Gessa G.L. (2012). Disulfiram stimulates dopamine release from noradrenergic terminals and potentiates cocaine-induced dopamine release in the prefrontal cortex. Psychopharmacology.

[33] Sheel-Kruger J., Randrup A. (1967). Stereotyped hyperactivity behavior produced by dopamine in the absence of noradrenaline. Life Sci..

[34] Reis D.J. (1972). The relationship between brain norepinephrine and aggressive behavior. Res. Publ. Ass. Res. Nerv. Ment. Dis..

[35] Rabkin J.M., Corless C.L., Orloff S.L., Benner K.G., Flora K.D., Rosen H.R., Olyaei A.J. (1998). Liver transplantation for disulfiram-induced hepatic failure. Am. J. Gastroenterol..

[36] Watts T.E., Pandey R.A., Vancil T.J. (2014). Fatal fulminant hepatic failure related to the use of disulfiram. J. Ark. Med. Soc..

[37] Nagendra S.N., Shetty K.T., Rao K.M., Rao B.S. (1994). Effect of disulfiram administration on rat brain glutathione metabolism. Alcohol.

[38] Ohno Y., Hirota K., Kawanishi T., Takanaka A. (1990). Loss of viability after disulfiram treatment without preceding depletion of intracellular GSH. J. Toxicol. Sci..

[39] Chen Y., Dong H., Thompson D.C., Shertzer H.G., Nebert D.W., Vasiliou V. (2013). Glutathione defense mechanism in liver injury: Insights from animal models. Food Chem. Toxicol..

[40] Swiderska-Kołacz G., Parka B., Kołątaj A., Klusek J. (2004). Wpływ morfiny i skopolaminy na zawartość glutationu i aktywność enzymów glutationowych w tkankach myszy. Med. Wet..

[41] Kong A.N., Owuor E., Yu R., Hebbar V., Chen C., Hu R. (2001). Induction of xenobiotic enzymes by the MAP kinase pathway and the antioxidant or electrophile response element (ARE/EpRE). Drug Metab. Rev..

[42] Atici S., Cinel I., Cinel L., Doruk N., Eskandrai G., Oral U. (2005). Liver and kidney toxicity in chronic use of opioids: An experimental long term treatment model. J. Biosci..

[43] Jalili C., Makalani F., Roshankhah S., Sohrabi K., Salahshoor M.R. (2017). Protective effect of resveratrol against morphine damage to kidneys of mice. Int. J. Morphol..

[44] Khairnar S.I., Mahajan U.B., Patil K.R., Patel H.M., Shinde S.D., Goyal S.N., Belemkar S., Ojha S., Patil C.R. (2020). Disulfiram and it copper chetlate attenuate cisplatin-induced acute nephrotoxicity in rats via reduction of oxidative stress and inflammation. Biol. Trace Elem. Res..

[45] Chahkandi M., Askari N., Asadikaram G. (2015). The effect of acute and chronic morphine on some blood biochemical parameters in an inflammation condition in gonadectomized male rats. Addict. Health.

[46] Nagi N., Murao T., Okamoto N., Ito Y. (2009). Disulfiram reduces elevated blood glucose levels in Otsuka Long-Evans Tokushima Fatty (OLETF) rats, a model of type 2 diabetes. J. Oleo Sci..

[47] Nagamatsu K., Ohno Y., Ikebuchi H., Takahashi A., Terao T., Takanaka A. (1986). Morphine metabolism in isolated rat hepatocytes and its implications for hepatotoxicity. Biochem. Pharmacol..

[48] Skoulis N.P., James R.C., Harbison R.D., Roberts S.M. (1989). Depression of hepatic glutathione by opioid analgesic drugs in mice. Toxicol. Appl. Pharmacol..

